# Following cytotoxic nanoconjugates from injection to halting the cell cycle machinery and its therapeutic implications in oral cancer

**DOI:** 10.1186/s12885-021-07849-x

**Published:** 2021-02-17

**Authors:** Hend M. Abdel Hamid, Zeinab E. Darwish, Sahar M. Elsheikh, Ghada M. Mourad, Hanaa M. Donia, Marwa M. Afifi

**Affiliations:** 1 Oral Pathology Department, Faculty of Dentistry, Alexandria University, Champilion Street, Azarita, Alexandria, Egypt; 2 Histology and Cell Biology Department, Faculty of Medicine, Alexandria University, Alexandria, Egypt; 3 Center of Excellence for Research in Regenerative Medicine and Its Application (CERRMA), Faculty of Medicine, Alexandria University, Alexandria, Egypt; 4 Clinical Pathology Department, Faculty of Medicine, Alexandria University, Alexandria, Egypt; 5 Laboratory of Cancer Biology and Genetics, Center for Cancer Research, National Cancer Institute, 37 Covent Dr., Room 4054, Bethesda, MD 20892 USA

**Keywords:** Gold nanoparticles, 5-flourouracil, Camptothecin, FGFR1 inhibitor, Oral cancer

## Abstract

**Background:**

The concept of personalized therapy has been proven to be a promising approach. A popular technique is to utilize gold nanoparticles (AuNPs) as drug delivery vectors for cytotoxic drugs and small molecule inhibitors to target and eradicate oral cancer cells in vitro and in vivo. Both drug and nanocarrier designs play important roles in the treatment efficacy. In our study, we standardized the nanosystem regarding NPs type, size, surface ligands and coverage percentage leaving only the drugs mode of action as the confounding variable. We propose that similarly constructed nanoparticles (NPs) can selectively leverage different conjugated drugs irrelevant to their original mode of action. If proven, AuNPs may have a secondary role beyond bypassing cancer cell membrane and delivering their loaded drugs.

**Methods:**

We conjugated 5- fluorouracil (5Fu), camptothecin (CPT), and a fibroblast growth factor receptor1-inhibitor (FGFR1i) to gold nanospheres (AuNSs). We followed their trajectories in Syrian hamsters with chemically induced buccal carcinomas.

**Results:**

Flow cytometry and cell cycle data shows that 5Fu- and CPT- induced a similar ratio of S-phase cell cycle arrest as nanoconjugates and in their free forms. On the other hand, FGFR1i-AuNSs induced significant sub-G1 cell population compared with its free form. Despite cell cycle dynamics variability, there was no significant difference in tumor cells’ proliferation rate between CPT-, 5Fu- and FGFR1i- AuNSs treated groups. In our in vivo model, FGFR1i-AuNSs induced the highest tumor reduction rates followed by 5Fu- AuNSs. CPT-AuNSs induced significantly lower tumor reduction rates compared with the 5Fu- and FGFR1i- AuNSs despite showing similar proliferative rates in tumor cells.

**Conclusions:**

Our data indicates that the cellular biological events do not predict the outcome seen in our in vivo model. Furthermore, our results suggest that AuNSs selectively enhance the therapeutic effect of small molecule inhibitors such as FGFR1i than potent anticancer drugs. Future studies are required to better understand the underlying mechanism.

## Background

The concept of cancer nanotechnology emerged as a hope to enhance chemotherapeutic drugs biodistribution, minimize off-target effects and enhance uptake in malignant cells [1]. Numerous studies reported the enhanced therapeutic effect against oral squamous cell carcinoma (OSCC) using a variety of nanoparticles, cytotoxic drugs and cancer models [2–5]. Accordingly, it is well accepted that nanocarriers act as vehicles to deliver drugs to their targets. For example, polyethylene glycol (PEG) coated nanocarriers play a critical role in overcoming opsonization and subsequent elimination from the blood stream [6]. Moreover, nanoparticles actively target oral cancer cell receptors such as surface integrins αvβ6 by peptides such as Arginyl-glycyl-aspartic acid (RGD) [7]. Both drug and nanocarrier designs play important roles in the treatment efficacy. Generally, the drug is designed to formulate compounds that inhibit cancerous cell proliferation, while the nanocarrier design aims to develop nano-vehicle structures that maximize drug concentration in the tumor relative to healthy tissue, thus reducing adverse drug effects [8]. We propose that similarly constructed nanoparticles (NPs) can selectively leverage different conjugated drugs irrelevant to their original mode of action. In our study, we standardized the nanosystem regarding NPs type, size, surface ligands and coverage percentage leaving only the drugs mode of action as the confounding variable. To confirm our hypothesis, we chose 5-fluorouracil (5Fu) and camptothecin CPT as cytotoxic drugs. Both drugs are used in treatment protocols of OSCC. Moreover, 5Fu and CPT interfere with DNA and RNA synthesis inhibiting S phase of the cell cycle via 2 different mechanisms. 5Fu is an antimetabolites cytotoxic drug, it interferes with nucleoside metabolism by incorporation into RNA and DNA molecules, leading to cytotoxicity and cell death. However, CPT is a plant alkaloid drug inhibits topoisomerase I enzyme activity required for DNA molecule integrity during replication [9, 10]. Then, we compared 5Fu and CPT cytotoxic effects to a small molecule inhibitor targeting fibroblastic growth factor receptor 1 (FGFR1). Following which, we synthesized average size (~ 30 nm) gold nanospheres (AuNSs) and sequentially coated their surface with ligands such PEG, RGD peptide, cytotoxic drugs 5Fu and CPT, or fibroblastic growth factor receptor 1 inhibitor (FGFR1i). This was performed using the same surface coverage ratio and chemical linker. At the end of the synthesis/conjugation phase, we assembled 3 nano-constructs, PEG-RGD-5Fu-AuNSs; PEG-RGD-CPT-AuNSs; and PEG-RGD-FGFR1i-AuNSs. Meanwhile, we chemically induced hamster buccal pouch carcinoma (HBPC) and injected the animals intraperitoneally with the corresponding nanoconjugates. We used the free forms of 5Fu, CPT and FGFR1i molecules as controls since their impact on cellular functions is well reported [11–14].

Following trajectories of the drug nanocarriers in our in vivo model, we found a heterogeneity in the underlying events leading to the final tumor reduction outcome. For example, 5Fu-AuNSs and FGFR1i-AuNSs had similar tumor reduction rates but different cell cycle dynamics. On the other hand, CPT-AuNSs and 5Fu-AuNSs had the same cell cycle dynamics but significantly different tumor volume reduction and global impact. Moreover, our findings show that conjugating a small molecule inhibitor, FGFR1i, to AuNSs induced the most favorable therapeutic outcome against OSCC compared with 5Fu-ANSs and CPT-AuNSs.This was rather surprising given how potent these drugs are even as free molecules. Our data suggests that AuNSs selectively enhance the therapeutic effect of small molecule inhibitors such as FGFR1i than potent anticancer drugs indicating that gold nanoparticles (AuNPs) play a greater role than only acting as cargo-carriers. If proven, AuNPs may have a secondary role beyond bypassing cancer cell membrane and delivering their loaded drugs.

## Methods

### Gold nanoparticles synthesis, characterization and functionalization

Using the citrate reduction method, we synthesized gold nanospheres (AuNSs, Chloroauric acid, Sigma Aldrich, 2,554,169, Germany) with an average diameter of 30 nm [15]. Briefly, 50 ml of 0.1% auric acid solution was heated while stirring until boiling. Then, 10 ml of 1% tri-sodium citrate was added with vigorous stirring. The solution continued to boil for 15 min to finally obtain the desired particle size/shape. To enhance AuNSs properties, we performed several surface modifications to functionalize the synthesized nanoparticles with the proper ligands.

Using a mono-layer percent coverage approach, we functionalized AuNSs with poly-ethylene glycol (PEG), a polymer used for further stabilization and to evade plasma protein opsonization in vivo [2, 6]. We added 1.0 mM solution of PEG 5000 MW (Sigma Aldrich, 1,546,580, Germany) dissolved in deionized water (diH_2_O) to AuNSs solution to achieve a molar ratio equivalent to 20% of surface coverage (ca. 565.56 mol of PEG were added per particle). Using the same approach, we further conjugated AuNSs with a custom peptide (RGD) that targets alpha and beta integrins expressed abundantly on the surface of OSCC cells. We dissolved 0.93 mM Arginylglycylaspartic acid (RGD, GenScript, USA Inc.) in diH_2_O which was immediately added to the PEG-AuNSs solution to achieve 25% surface coverage (ca. 706.95 mol of RGD were added per particle). Finally, we divided the PEG-RGD-AuNSs solution into 3 batches, where we further conjugated the drugs to the AuNSs via a pH-sensitive hydrazone linkage by methyl thioglycolate and hydrazine using a previously published method [16]. Final nano-conjugates were as follows, PEG-RGD-5FU-AuNSs, PEG-RGD-CPT-AuNSs, and PEG-RGD-FGFR1i-AuNSs. To establish this, we added 1.0 mM solution of 5FU, CPT (Selleckchemicals, S2045, S1288, respectively), or PD173074 (Sigma Aldrich, P2499, Germany) separately to PEG-RGD-AuNSs to accomplish a molar ratio equivalent to cover 55% of the surface (ca. 1555.29 mol of the drug were added per particle). For the EGFR1-i, we dissolved the inhibitor in dimethyl sulfoxide (DMSO) by 10 mg/ml. Then 100 μl from the working solution was dissolved in 1.9 ml glycrol according to the manufacturing protocol.

To make sure that our synthesis and functionalization processes were successful, we used transmission electron microscopy (TEM, JOEL, 1400plus, Japan) and the Nano-Zetasizer (Malvern Instruments, Worcestershire, UK) to confirm the size and morphology of the synthesized AuNSs. We took measurements before, during, and after conjugation to compare changes in size and surface charges. This is critical, since the interaction of AuNSs with the biological environment and biocompatibility depends on their surface charge [17]. We also measured the absorption spectra of the different nanoconjugates using a UV-VIS spectrophotometer (The Thermo Scientific™ Evolution 300, USA) at 530 nm to quantify the distribution of AuNSs.

After successfully synthesizing the nano-constructs, we wanted to confirm that the chemical linker joining the drugs to AuNSs was pH- sensitive. This is important because when the particles get taken up by cells, the acidic pH in the lysosomes breaks the chemical linker and induces drug release. To measure pH sensitivity, we added an acidic buffer solution (pH=5) to the nanoconjugates solutions and allowed them to shake for 5 mins at 37 °C. After which the solutions were centrifuged for 10 min at 6000 rpm. The absorbance peak of the supernatant was measured using a UV-VIS spectrophotometer (DeNovix DS-11 FX +). If the linker is pH-sensitive, the absorbance peak equivalent to each drug should be seen, as previously reported [16].

### Animal model

We conducted a controlled comparative experimental study using 120 Syrian golden male hamsters (*Mesocricetus auratus*, 5 weeks old, weighing 80–110 g). They were obtained from VACSERA, Cairo, Egypt. The animal study was approved by the Alexandria University review committee and the procedures followed are in accordance with institutional guidelines (IRB#00010556-IORG0008839). The hamsters were weighed once per week throughout the whole period of the experiment. They were housed in show box cages (Technoplast, Italy) one per box under the same condition on a regular alternating lighting cycle (12:12 light: dark).

To establish an oral cancer model, we chemically induced OSCC by painting the left buccal pouch, only to facilitate eating by the other side, of the hamsters with 7, 12 dimethylbenz [a] anthracene carcinogen (DMBA, Sigma Aldrich, 57,976 Germany). We used hamster buccal pouch as our oral cancer model due to the similarities between its lining mucosa and the epithelium covering hard palate, tongue and gingiva of human oral cavity. Furthermore, multiple correspondence to human OSCC were found regarding morphology, molecular markers expression, and finally DNA mutations [18].

During the carcinogenesis phase, we used DMBA along with a carbamide peroxide as a promoter, 5 days per week (alternate days, 3 days for DMBA and 2 days for the promoter) [19]. We specifically used this promoter to decrease the induction period from 16 weeks to less than 12 weeks and obtaining a well-developed intraoral OSCC exophytic masses. This was done to minimize the handling procedures throughout the experiment with the animals and the carcinogen. Carcinogenesis was evaluated microscopically 4 weeks after induction by sacrificing cohorts of 2 hamsters every 2 weeks and evaluating lesions with H&E staining until well-established OSCCs were detected. Moreover, hamsters with tumor size greater than 2 cm in diameter were dropped from the study and euthanized before the predetermined time point (4 weeks).

### Treatment protocol

After inducing visible oral exophytic polyps, the reminder hamsters (*n*=104) were blindly randomized into 8 groups using computer generated list of random numbers (*n*=13 hamster per group). Three groups were injected with the free forms of 5Fu, CPT and FGFR1i with a dose of 12, 2, and 0.5 mg/kg, respectively [20, 21]. Another 3 groups were similarly injected up to 150 μl of the prepared functionalized AuNSs with the 3 drugs 5Fu-AuNSs, CPT-AuNSs, and FGFR1i-AuNSs equivalent to the administrated free dose of 5Fu, CPT and FGFR1i (12, 2, and 0.5 mg/kg), respectively. Finally, 2 groups served as controls receiving saline and PEG-RGD-AuNSs (150 μl same as treatment dose) without any loaded drug. Each group received the designated treatment 3 times/week for a period of 1 week by an intraperitoneal (I.P.) injections under lightly sedation using ketamine hydrochloride [22]. All investigators were blinded throughout the whole experiment (During allocation concealment, outcome assessment and data analysis). The animal groups were known only by the animal care staff who administered the drugs. After treatment according to the planned protocol, animals were sacrificed by cervical decapitation under anesthetic conditions (Ketamin 30 mg/kg, i.p.). Finally, any animal disposals were burned.

### Tumor volume reduction rate and survival analysis

We measured tumor volume before and weekly after (be more specific) administration of the treatment. It was estimated by using the formula (D max X D min^2^/2), where (D max) represents longer dimension and (D min) represent shorter dimension. We performed sequential measurements over a period of 4 weeks to assess the tumor volume percentage change and we calculated the survival rate as days.

### Drug release and localization upon cellular uptake

We tested the cellular uptake of AuNSs and subsequent release of the drugs using confocal laser scanning microscopy (CLSM, Leica TSC SPII/DMi 8). We used a previously published method to confirm drugs release [16]. Since 5Fu and CPT have fluorescence properties (emission spectrum: 405 nm and 490 nm, respectively), we were able track their release and localization within cancer cells. Moreover, no nuclear fluorochrome was applied to tissue sections treated with 5Fu-AuNSs, CPT-AuNSs, free 5Fu and CPT to avoid misinterpretation with the blue fluorescent for of CPT drug. But since EGFR1i does not possess fluorescence emission signal, we stained the targeted receptor using anti-FGFR1 antibody (Abcam, ab10646, USA) at a concentration of 1:100, together with its compatible 2^ry^ antibody Alexa Fluor 488 (Abcam, ab150077, USA). Hoechst 33342 (Sigma Aldrich, 23,491–52-3, Germany) was used as a counterstain for DNA staining. After scarification procedures, we dissected the tumors biopsies and divided each one into 2 equal specimens. We selected the central part of tumoral tissue for all histologic evaluation. We examined 5 different histologic sections by 2 different pathologists for each slide on a magnification power × 63. For autofluorescent cytotoxic drugs, we quantified the differences in the nuclear signals between free and conjugated counterparts using ImageJ software (version 1.52p).

### Histologic and immunohistochemical evaluation

We stained the tumor tissue sections with H&E stain to visualize the presence of oral carcinomas. To test the proliferative index of the tumor cells, we stained tissue sections using proliferating cell nuclear antigen (PCNA, Thermo scientific, MS-106-R7, USA) with a 1:50 dilution ratio of mouse monoclonal anti-PCNA antibody. We also used Ki67 to confirm the PCNA staining results (Ki67, Thermo scientific, # MA5–14520, USA) with a 1:200 dilution ratio of rabbit monoclonal anti-Ki67 antibody. We assessed tumor proliferation using different markers to capture all growing fraction in the tumor especially Ki67 which is steadily expressed throughout all cell cycle phases except G0 [23]. Immunohistochemical (IHC) staining was performed using the labeled streptavidin-biotin complex method (LSAB) [24] and the stained slides were captured by Motic image plus 2.0. PCNA and KI67 nuclear staining were measured by calculating intensity as mean area percent (MA%) using ImageJ software (version 1.52p). Sections were blindly examined by 2 pathologists in randomly 5 selected microscopic fields at a magnification of × 400.

### Cell cycle analysis

We preserved a part of the excised oral tumors in complete RPMI tissue culture media (Sigma Aldrich, R8758, Germany). Following a modified standard protocol [25], we homogenized the fresh tissue specimens by thoroughly mincing with sharp surgical blades in cold RPMI medium on disposable petri dishes. The released cells were separated from remaining tissue by 100 μm nylon cell strainers, centrifuged (2000 rpm, 20 min), and incubated with 1 ml trypsin enzyme for 20 min. The cells were centrifuged again (2000 rpm, 5 min) to remove the trypsin enzyme. Afterwards, 2 washes were done using FACS buffer (PBS+ BSA) followed by centrifugation for 5 min after each wash. Then, we fixed the cells using 70% ice cold ethanol while vortexing to prevent cell clumping and stored the cell suspensions at − 20 °C until FACS analysis. Upon examination, the cell suspension was allowed to reach room temperature and centrifuged for 5 min to remove excess alcohol. This was followed by 2 washes using FACS buffer with centrifugation for 5 min after each wash. Further, sample purification was done by re-filtering the cell suspension using 100 μm nylon cell strainers mesh. Finally, we added 100 μg/ml of propidium iodine PI (Sigma Aldrich, P4864, Germany) for DNA staining and 200 μg/ml RNAse (Sigma Aldrich, R5500, Germany) for RNA digestion at room temperature for 15 min. We analyzed all the samples using a FACS Caliber (BD Biosciences, USA).

### Evaluation of the systemic adverse effects of different treatments

We monitored the hamsters for signs of alopecia, diarrhea and weight loss throughout the treatment course. To investigate the impact of these nano-constructs on the hematopoietic system since this is one of the most affected organs after treatment, we collected blood samples from all the hamsters to assess possible myelosuppression. After administration of the final treatment dose, we sedated the hamsters to collect blood samples from the retro-orbital venous plexus [26]. The samples were taken at fixed intervals of 1, 24 and 48 h, 1 week and at time of euthanasia (4 weeks). We assessed the count of red blood cells (RBCs), white blood cells (WBCs), hemoglobin, and platelets. At necropsy, we examined the rest of the buccal pouch dissected from hamsters for any signs of drugs related effects. Moreover, we collected the liver and kidneys to histologically identify any damage to their normal architecture. The tissue specimens were fixed in 10% neutral-buffered formalin solution and embedded in paraffin wax for H&E staining and examined with a light microscope.

### Data representation and statistical analysis

Data analysis and graphs were produced using GraphPad Prism software (Prism 8, version 8). All values were expressed as mean ± standard deviation (SD). Tumor volume change, IHC staining data, nuclear localization data were analyzed using one-way ANOVA test and further analysed using Dunnett’s multiple comparison tests. Blood count was analyzed using two-way ANOVA test to include 2 factors of interest, time and treatment. Hamster survival curves were estimated by Kaplan–Meier method and analyzed by the Log-rank (Mantel- cox) test. The level of statistical significance (*p*< 0.05) was indicated on plots with asterisks (*). We used model assumptions checking using the Shapiro-Wilk normality test and Levene’s test for homogeneity of variance. The sample size calculation was based on the expected difference in the cell cycle counts between the group treated with 5Fu as free form against the conjugated form =40%, with precision of 10% using alpha error = 0.05 with study power of 80%. The sample size was calculated using G.STATA 11 Software. The total sample size was calculated as 112 hamsters then increased to reach 120 hamsters to compensate sample drop off (death as a complication of the cancer induction) [27].

## Results

Using HBPC as our cancer model, we noticed a generalized increased potency of all the conjugated drugs compared with their free form with almost no off-targets side effects. Interestingly, we found that AuNSs selectively enhances the therapeutic effect of small molecule inhibitors such as compared with potent anticancer drugs such as 5Fu and CPT.

### Characterization and functionalization of the synthesized AuNPs

**Fig. 1:**
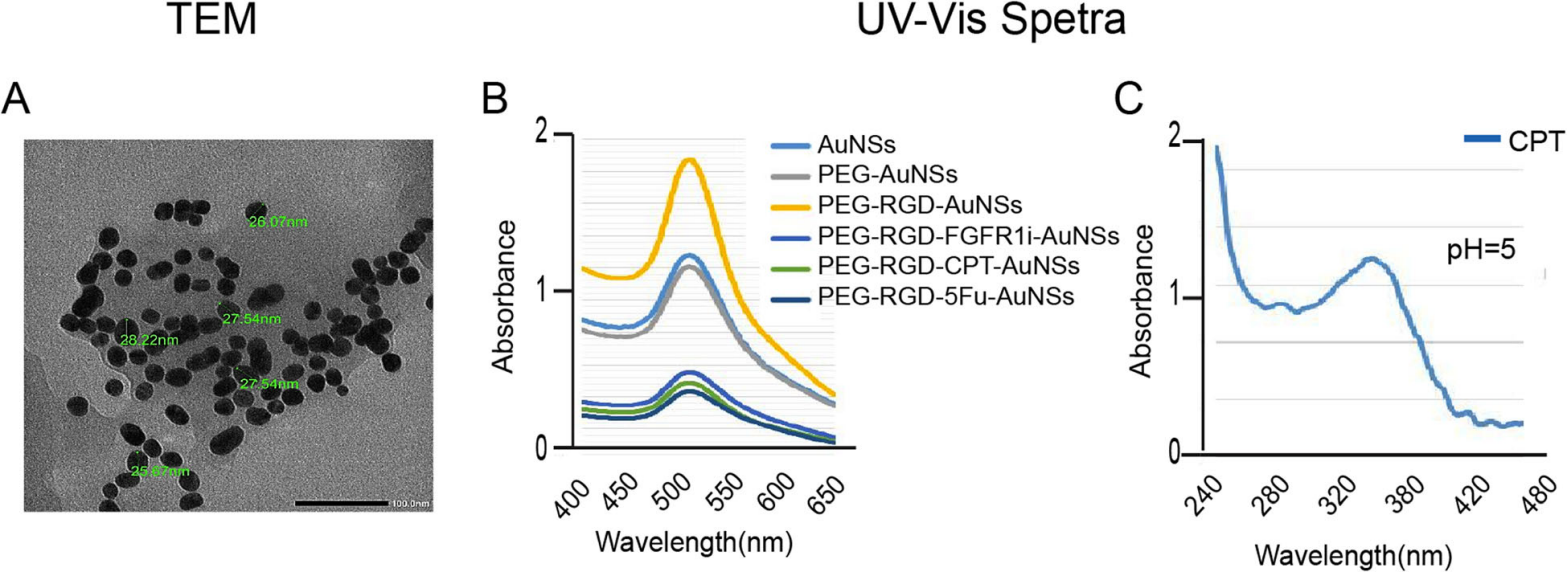
Characterization of the synthesized AuNSs. **a** TEM image revealing a monodispersed spherical nanoparticle solution with an average size of 27.1 nm. **b** UV–VIS optical absorption spectra confirming the size of the AuNSs with the subsequent conjugations with a peak equal to 528 nm (scale bar 50 nm). It shows that no peak shift indicates that no clumping or aggregation of the nanoparticles occurred after each ligand conjugation. **c** UV-Vis spectra of CPT nanoconjugates at pH=5 with a peak at 340 nm which corresponds to free CPT being released from AuNSs at acidic pH

Synthesized AuNSs were characterized to verify their size, shape and mono-distribution. Using TEM, and the Nano-Zetasizer, we confirmed that the synthesized AuNSs were monodispersed spherical nanoparticles with a mean size of 27.1 ± 2.73 nm (Fig. 1a). UV-Vis spectrometer showed a peak at maximum absorbance equal to 528 ± 1 nm, which is equivalent to an average size of 30 nm. There was no change in the maximum absorbance of the nanoconjugates (~ 528 nm) detected by the UV-spectrometer, indicating lack of aggregation with no significant change in size (Fig. 1b) [28].

To verify the conjugation process, we used the Nano-Zetasizer and UV-spectrometer to take measurements before and after loading each ligand. The changes in the surface charge of nanospheres, measured by the Nano-Zetasizer, reflected the docking status of the nanospheres. For example, bare AuNSs solution was strongly negative (− 24 mV), indicating a stable colloidal AuNPs solution with no possibility for subsequent aggregation [29]. Coating AuNSs with different ligands induced changes in zeta potential measurements as follows: − 24 mV to − 26.6 mV when adding PEG followed by − 21.3 mV upon RGD conjugation. Finally, − 19.7 mV was the zeta potential value of the AuNSs suspensions after adding 5Fu, CPT, or FGFR1i which was measured after conjugation and immediately before IP injection (Table 1). The change noted in the zeta potential values indicated a successful conjugation process [30]. We previously reported that AuNSs loaded with drugs release their cargo upon cellular uptake in the lysosomes due to the acidic pH (pH=5) [29]. Accordingly, we wanted to confirm that the hydrazone bond used to conjugate AuNSs linkage in the synthesized nanoconjugates are pH-sensitive. Therefore, we did an in vitro experiment in a test tube, where we added an acidic buffer to the nano-constructs. After centrifugation, we discarded the AuNSs pellet and measured the absorbance of the supernatant. After adding an acidic buffer to the nanoparticles solution, UV-VIS results showed peaks representing the drugs released in the acidic pH something. Figure 1c shows an example of an absorbance spectrum of the supernatant separated from CPT-AuNSs solution. Note the peak was located around 360 nm, which is equivalent to the maximum absorbance of CPT as reported previously [31]. The UV-Vis results verified that acidic pH released the drugs from their nanocarriers.

**Table 1:** Zeta potential readings

AuNSs	Zeta potential (mV)^a^
AuNSs	− 24.6
PEG-AuNSs	−26.6
RGD-PEG-AuNSs	−21.3
5FU-AuNSs	−19.3
CPT-AuNSs	−12.8
FGFR1i-AuNSs	−6.47

^a^mV stands for millivolts

### Tumor penetration and cellular uptake of the nanoconjugates

We investigated tumor and cellular uptake, and delivery of different nano-constructs in comparison with their free forms. Our research group recently performed an extensive characterization to verify NPs uptake, in vitro, of a similar system but with Doxorubicin as the cytotoxic agent of choice [32]. Here in, we used the fluorescence properties of CPT and 5Fu to quantify the cellular uptake of the nanoconjugates in comparison to the free form. Based on a previously published principle, we used the selective quenching effect induced by the plasmonic field of AuNSs to track tumor penetration and cellular uptake in our OSCC animal model [29].

**Fig. 2:**
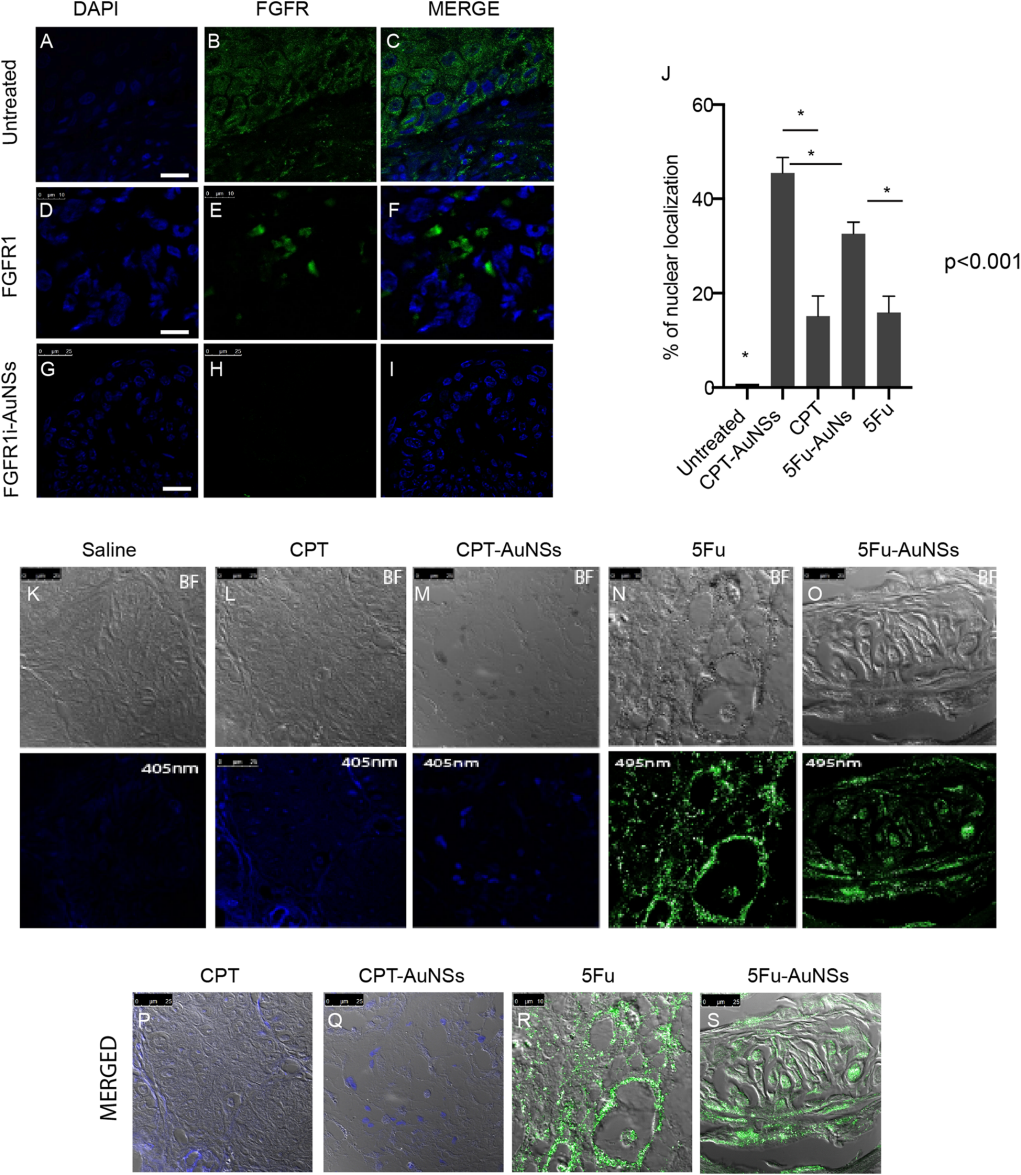
Confocal images of tumor tissue sections showing intra-tumoral localization of FGFR1i- 5Fu- and CPT-AuNSs. **a-c** Untreated tumor tissue sections exhibited strong and diffuse fluorescent signal indicating strongly expressed FGFR1. **d-f** Tumor tissue sections treated with free-FGFR1i show decreased FGFR1 expression compared with control indicating successful tumor penetration and inhibition of FGFR1. **g-i** Tumor tissue section treated with FGFR1i-AuNSs shows total loss of FGFR1 expression as a function of enhanced FGFR1i-AuNSs tumor penetration and selective targeting. **k** Untreated tumor tissue section lacking any fluorescence signal. **l**, **n**, **p**, **r)** Tissue sections showing molecules of CPT and 5Fu localization on tumor cell plasma membranes rather than the nuclei. **m**, **o**, **q**, **s** Tissue sections treated with CPT-AuNSs or 5Fu-AuNSs showing drug localization within the nucleus, thus conjugating 5Fu and CPT to AuNSs enhanced tumor penetration and increased cellular uptake (CPT and 5Fu fluorescence emission at 405 nm and 495 nm, respectively). No nuclear fluorochrome was applied. Magnification × 63, Scale bar =25 μm except (M), which is 10 μm. **j** Percentage of nuclear localization denotes that 5Fu- and CPT- AuNSs significantly enhance nuclear accumulation by 2-fold and 3-fold, respectively; CPT-AuNSs shows marked intense intranuclear staining compared with 5Fu-AuNSs *p*< 0.001. (*n*=13)

To study the extent of tumor penetration of FGFR1i-AuNS, we stained the treated tumor tissue sections with anti-FGFR1 biomarker because FGFR1i molecules lack autofluorescence properties. This approach allowed us to trace the FGFR1i inhibitory effect as a function of its tumor penetration and selective targeting of cancer cells. Untreated tumor tissue sections showed a generalized and diffuse cytoplasmic reaction throughout the specimen, reflecting high expression levels of FGFR1 (Fig. 2a-c). Tissue sections isolated from tumors treated with FGFR1i revealed a lesser extent of FGFR1 expression, mostly towards the periphery of the tumor (Fig. 2d-f). We noted complete loss of FGFR1 expression in tumors treated with FGFR1i-AuNSs, extracted from the center of the specimen; thus, indicating a high tumor penetration and enhanced drug delivery to cancer cells (Fig. 2g-i)**.**

Cytotoxic drugs such as 5Fu and CPT are commonly used to treat OSCC in vitro, in vivo*,* and in patients. They possess a fluorescence emission signal, which can be used to quantify the extent of their penetration in the tumor and their nuclear localization upon cellular delivery. Our data reveals that conjugating 5Fu and CPT to AuNSs significantly enhanced tumor cell uptake and more specifically nuclear localization by at least 25% compared with the free forms (Fig. 2k-s). Tumor specimens treated with 5Fu-AuNSs and CPT-AuNSs showed an intensified fluorescent signal located in the nuclei, which became less prominent towards the cell membranes of tumor cells as shown in (Fig. 2m, o, q, s). This can be explained by intra-nuclear drug accumulation after their release from AuNSs in the lysosomes. Meanwhile, the free forms of the drugs were able to passively penetrate the tumor tissue, compared with untreated tumor section, and accumulate in the plasma membrane of tumor cells showing much less colocalization in the nuclei (Fig. 2l, n, p, r**).** 5Fu and CPT along with their nanoconjugates revealed a significant intranuclear drug-nanoconjugate accumulation compared to their free forms by a 2-fold increase for 5Fu and 3-fold for CPT. Although 5Fu and CPT free forms resulted in same fluorescent signal values, however, upon calculating the mean percentage of CPT-AuNSs and 5Fu-AuNSs nuclear localization, we discovered a significant increased fluorescent signalling for CPT-AuNSs as shown in (Fig. 2j) compared to 5Fu-AuNSs.

### Cell cycle analysis

After fluorescence imaging confirmed superior penetration and drug delivery in tumors treated with the nanoconjugates, the next critical milestone was looking into cell cycle progression anomalies. This is because targeted signaling pathways and proteins induce a direct impact on cell cycle progression, regardless of the upstream events leading to it.

**Fig. 3:**
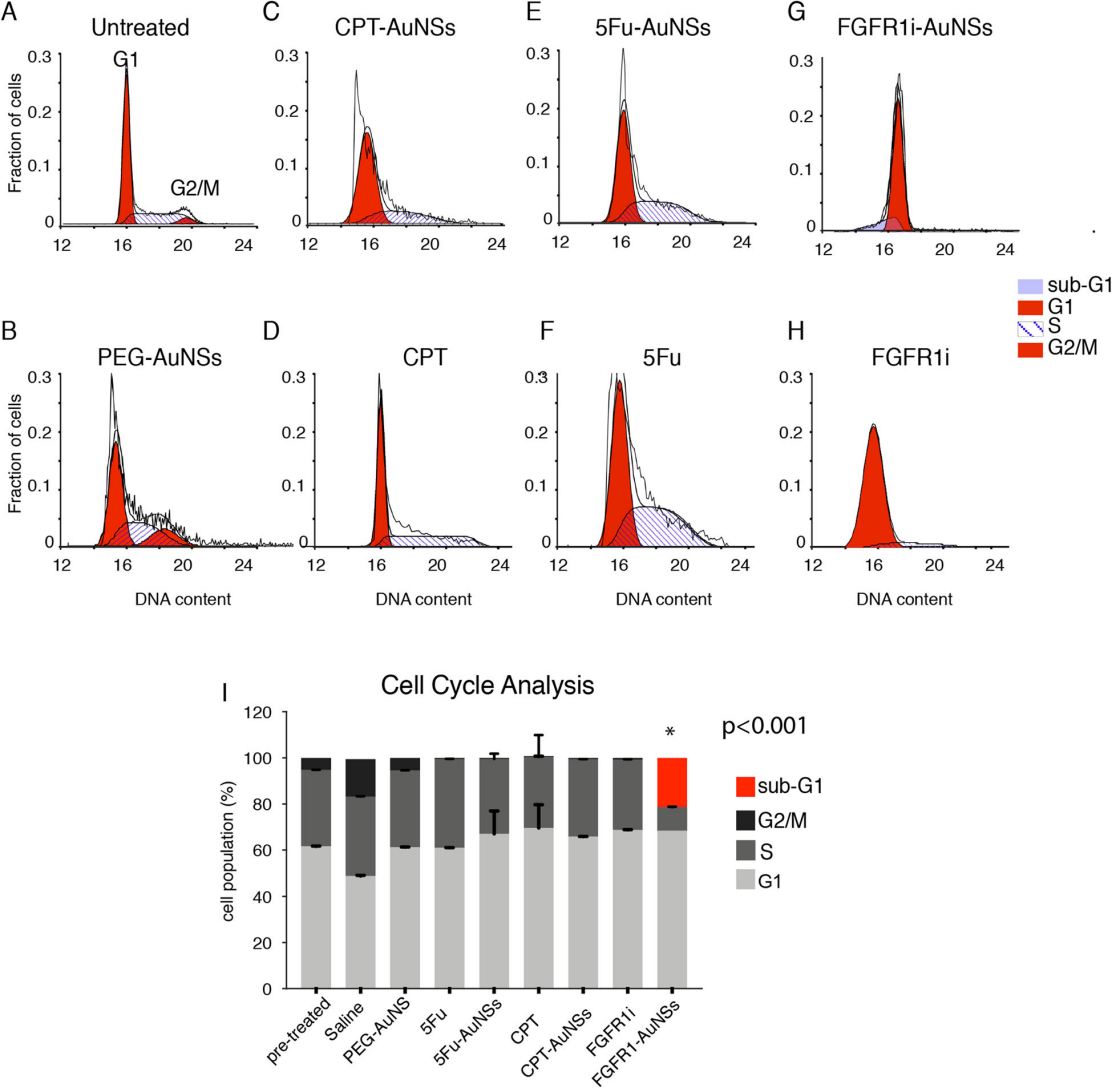
Cell cycle analysis in different study groups 4 weeks post-treatment compared with pre-treatment analysis. **i** Notice the G1 arrest in all study groups with a significant subG1 apoptotic population detected only in FGFR1i-AuNSs. (*n*=13)

Cell cycle analysis have shown that tumor cells isolated from animals treated with 5Fu or CPT, whether in their free form or conjugated to AuNSs, have similar distribution of cell cycle phases at a given time point after treatment. The cytotoxic drugs induced S-phase arrest (40% of the cell population) with complete depletion of G2/M. Tumor cells that were in G1 phase comprised around 60% of the total population (Fig. 3). Cancer cells isolated from tumors treated with free FGFR1i or its conjugated form also showed complete G2/M depletion, while maintaining the same percentage (~ 60%) of G1-phase cells as those treated with 5Fu and CPT drugs. Interestingly, ~ 22% of tumor cells treated with FGFR1i-AuNSs were in subG1-phase, which is indicative of an activated cellular apoptosis and subsequently cell death (Fig. 3) [33].

### Cessation of tumor cells proliferation

Our cell cycle analysis shows that the drugs conjugated to AuNSs had similar cell cycle phase distributions with the exception of FGFR1i-AuNSs which induced a 22% of the cancer cells to arrest in sub-G1(G0). Changes in the cell cycle phase distributions are induced by the drug’s mechanism of action and that AuNSs are indeed vehicles. We then wanted to determine what happens once cell cycle progression is disturbed. Therefore, we used anti-PCNA and anti-Ki67 biomarkers to assess the proliferative activity and cell cycle progression of the tumor cells in different groups. PCNA is a nuclear nonhistone protein that is necessary for DNA synthesis, thus higher expression levels are noted during G1-S phase transition [34]. Ki67 protein expression levels peak at G2 and G2/M phase [35]. Tumor tissue sections showed numerous nests of malignant epithelial cells in fibrous tissue stroma. Each nest is peripherally lined with columnar basal cells, while the center is formed of polyhedral-like cells. Several nests showed central keratin pearls indicating that excised polyps are mostly moderate well differentiated OSCC.

**Fig. 4:**
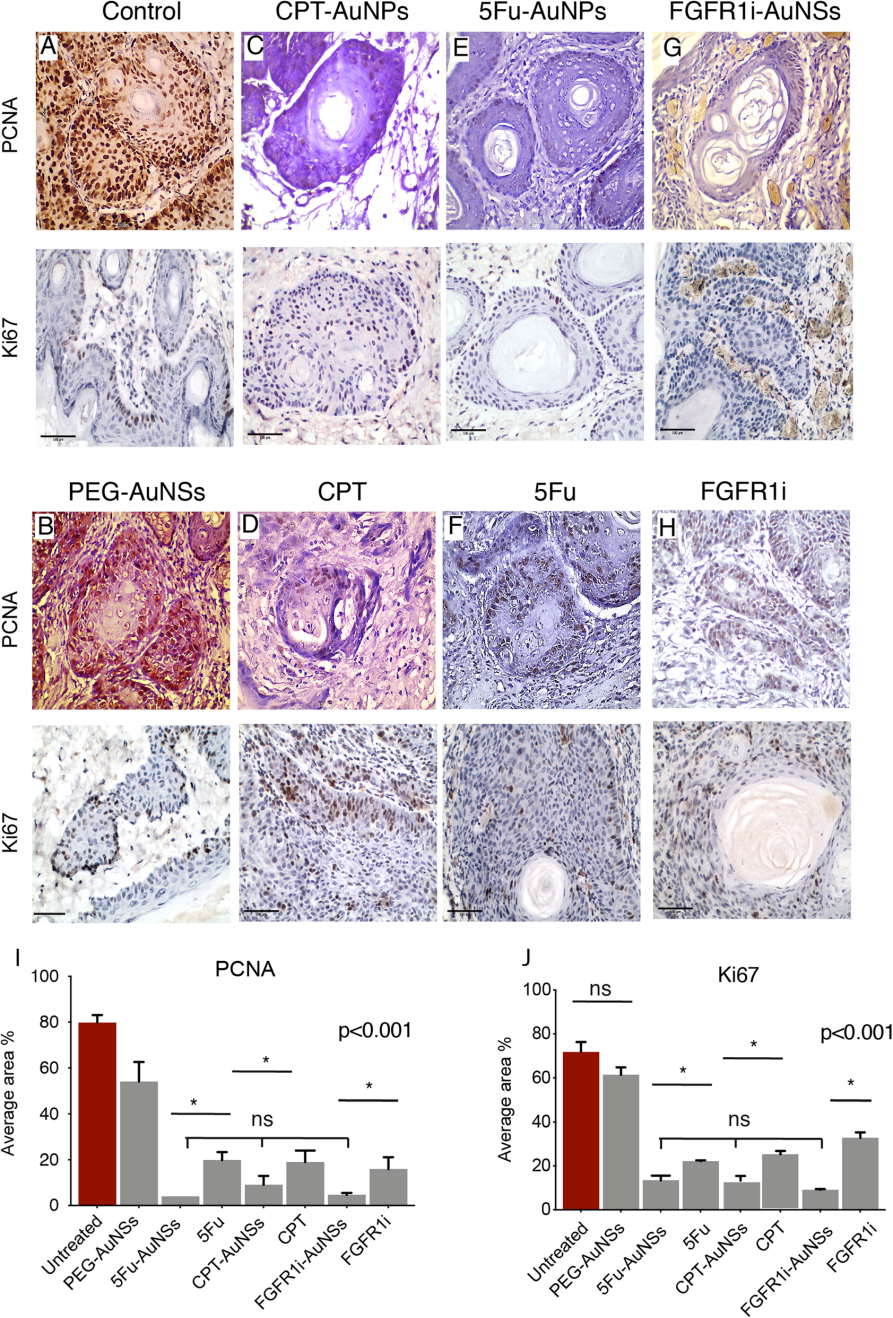
Immunohistochemical expression of PCNA and Ki67 in tumor tissue sections isolated from treated animals. **a**, **b** Untreated tumors cells expressing PCNA and Ki67 proteins indicating their cycling status, while PEG-AuNSs seems to induce a minimal inhibition to PCNA and Ki67 proteins. **c-h** FGFR1i- CPT- and 5Fu- AuNSs induced a significant inhibition to PCNA and Ki67 activity compared with the free form. **i**, **j** Bar chart plots of the average tissue section area showing positive immunoreaction for both PCNA and Ki67 normalized to the total area. The drugs and their corresponding nanoconjugates resulted in significant lack of proliferation of the tumor cells compared with untreated and PEG-AuNSs treated cells. There was no significance difference between the inhibitory effect induced by CPT, 5Fu and EGFRi (denoted by ns). Also, the same drug nanoconjugates appear to induce equivalent rates of cellular arrest. PCNA and Ki67 immunoreactions were significantly lower in tumor cells treated with FGFR1i- CPT- and 5Fu- AuNSs compared with the free forms. Findings were considered statistically significant when *p*< 0.001 and are denoted by asterisks. Scale bars=100 um (*n*= 13)

Untreated tumors were comprised of malignant cell nests that showed positive PCNA and Ki67 immuno-expression. Cell nests showed diffuse positive nuclear PCNA immunoreaction, while Ki67 staining was only localized in the nuclei of the basal cells (Fig. 4a). PEG-AuNSs treated tumor sections showed a humble negative impact on the proliferative rates of tumor cells (Fig. 4b, i, j). Tumors treated with nanoconjugate drugs showed significantly less PCNA and Ki67 immunoreaction (< 20% of the total area occupied by tumor cells) compared with those treated with the free form of the drugs (< 30%), but both forms induced significant decrease in the proliferative capacity of the tumor cells compared with untreated tumors (> 70% of the total area occupied by tumor cells) (Fig. 4a-j). We found that CPT, 5Fu, and FGFR1i which have different mechanism of actions inhibited the proliferative activity of tumor cells in a similar rate (Fig. 4i, j).

### Impact of FGFR1i-, CPT-, and 5Fu-AuNSs injections on the developed HBPC

By the end of the cancer induction phase, 10 hamsters with hepatosplenomegaly and ascites died as a complication related to ingestion of DMBA painting. Six hamsters were scarified, and buccal tissue biopsies were fixed and stained with H&E to evaluate the carcinogenesis. The remaining 104 hamsters developed polypoid OSCC tumor masses with an incident rate of 100% and tumor multiplicity of 1.27 tumors per hamster. Microscopically, the developed carcinomas were in the form of well, moderately and poorly differentiated types resembling human oral carcinomas with chronic inflammatory cell infiltrates in the supporting connective tissue.

**Fig. 5:**
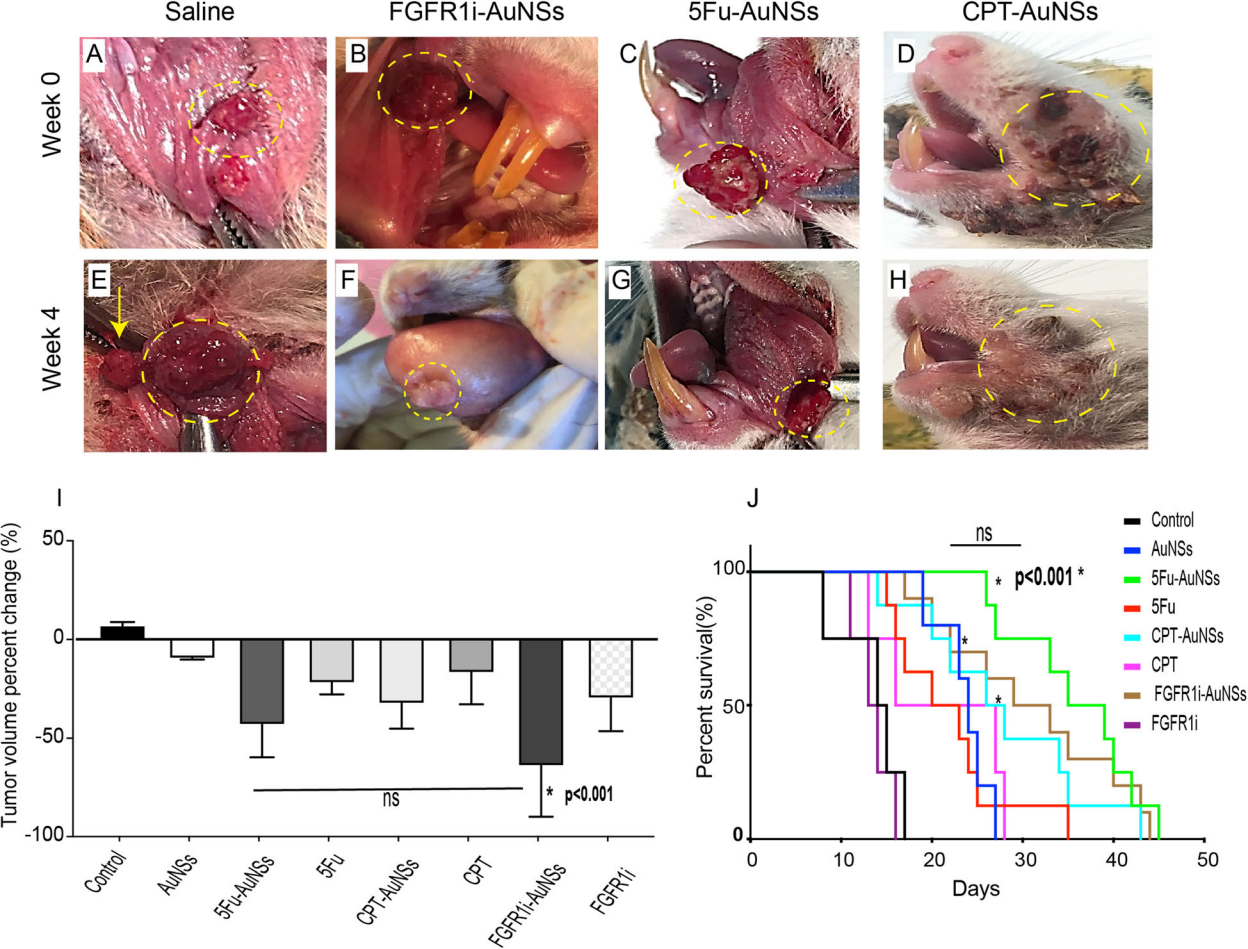
Clinical follow up after different treatments. Tumor volume measurements were conducted at week 0 and week 4 post-treatments. Note lesions within the yellow dashed circles. **a-d** Intra and extra oral tumors at week 0 just before treatment with saline, FGFR1i-AuNSs, 5Fu-AuNSs and CPT-AuNSs, respectively. **e** A hamster treated with saline showed a significant increase in tumor volume, at 4 weeks post-treatment. Notice the formation of new intraoral exophytic polyps (yellow arrow). **f** A hamster treated with FGFR1i-AuNSs showed enhanced tumor shrinkage with decreased vasculature. **g**, **h** Animals treated with 5Fu-AuNSs and CPT-AuNSs also showed reduction in tumor size. **i** Tumor volume percentage change across the 8 groups showing enhanced tumor reduction rate related to FGFR1i-AuNSs treatment (− 63.09%, *p*< 0.001), followed by 5Fu-AuNSs (− 43.4%, *p*< 0.001). There was no significant difference between the 2 treatments indicating a favorable clinical effect (ns). **j** Kaplan Meier survival curve demonstrating significantly increased life span in animals treated with 5Fu-AuNSs, FGFR1i-AuNSs and CPT-AuNSs compared with control and free form of the drugs, denoted with asterisks (*p*< 0.001). Note the lack of significant difference among the nanoconjugates (ns). (*n*= 13)

To test the therapeutic efficiency of the nanoconstructs, we measured tumor volume in different animal groups over time **(**Fig. 5a-h**)**. After 4 weeks of treatments, animals treated with FGFR1i-AuNSs yielded the highest reduction in tumor volume with a 2-fold decrease (− 63.09%) compared with those injected by CPT-AuNSs (− 32.1%). Animals treated with 5Fu-AuNSs showed a tumor reduction rate equal to − 43.4%. There was no significant difference in tumor reduction rates induced by 5Fu- and FGFR1i- AuNSs (*p*> 0.05) (Fig. 5i).

Several factors affect an animal’s life span during cancer treatment. Tumor burden and toxicities related to therapeutic administration are considered the most important factors [36]. We measured survival rates across animal groups to assess the impact of the nanoconjugates versus free drugs on survival. The control group receiving saline showed an average survival of 12.4 days after the final dose. Similarly, animals treated with free FGFR1i showed poor survival rate (13.5 days). We noted that the free forms of CPT, 5Fu, and PEG-AuNSs enhanced the survival rates reaching 21and 24 days post treatment. This was significantly higher than control (*p*< 0.001). Conjugating AuNSs with FGFR1i, 5Fu, and CPT significantly enhanced survival rates to more than 27 days post treatment. Notably, the difference in life span among the 3 nanoconjugates was not statistically significant (Fig. 5j).

### Off-target systemic effects

**Fig. 6:**
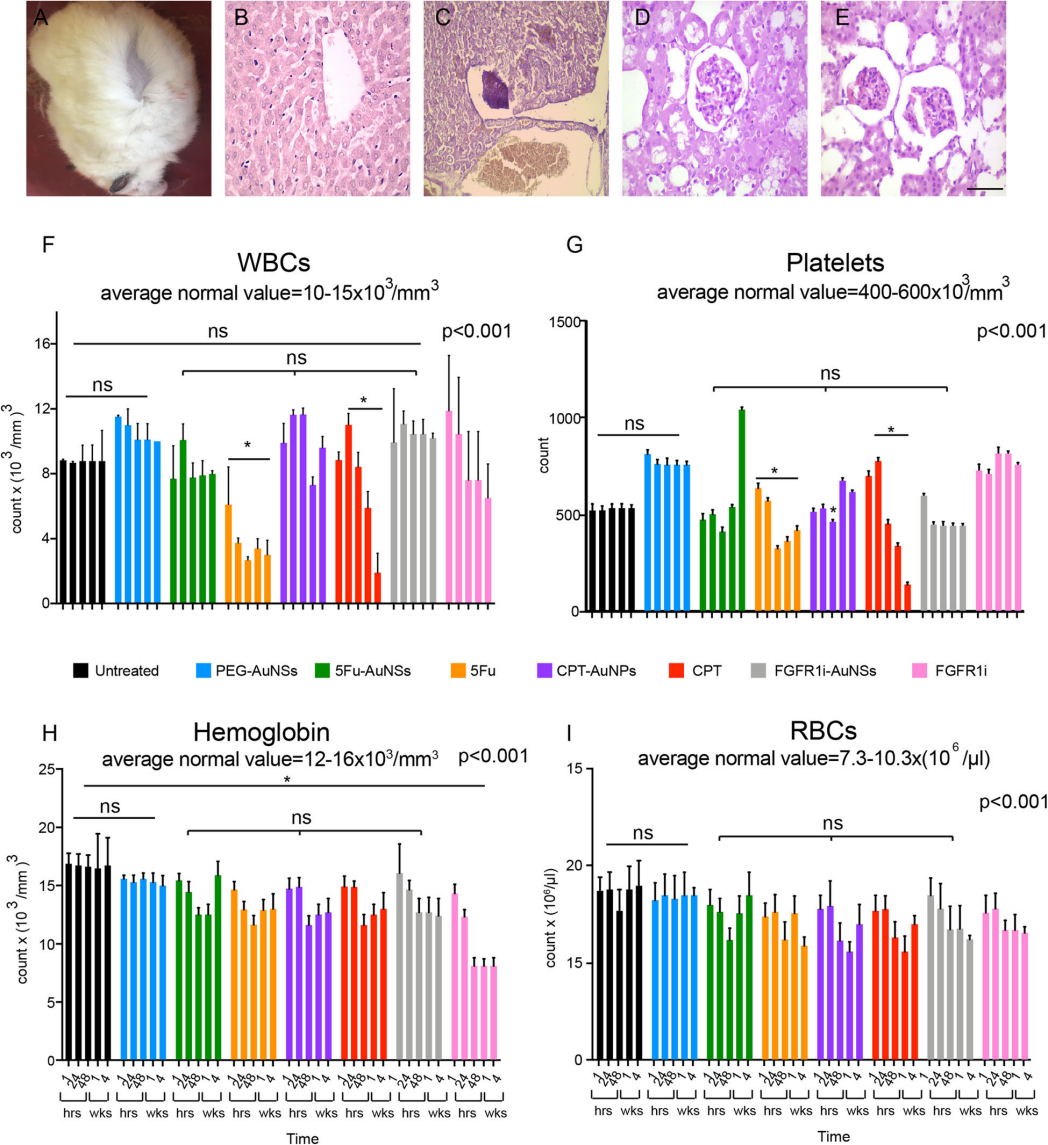
Off target different systemic effects. We conducted clinical, histopathologic and hemopoietic assessment 4 weeks post-treatment. **a** A hamster treated with CPT showing partial alopecia. **b** Normal liver section extracted from 5Fu-AuNSs conjugated group showing normal hepatocytes and liver parenchyma. The same images were obtained from the other 2 nanoconstructs. **c** Liver section showing focal necrosis near the portal vein obtained for both free cytotoxic drugs CPT and 5Fu. **d**, **e** Areas of glomerular shrinkage in hamsters receiving CPT and 5Fu. **f** Platelet count for the 8 groups across all time points; no significant difference was noted between the 3 nanoconjugates groups (ns) with only one exception seen for CPT-AuNSs at 48 h. A significant decrease in the platelets count was seen in both free 5Fu and CPT denoted with asterisks (*p*< 0.001). **g** RBCs count showing no significant difference between the 3 nanoconjugates groups (ns) (*p*< 0.001). **h** WBCs values also show no significant difference between the 3 nanoconjugates groups (ns) (*p*< 0.001), while a significant fluctuation was seen for WBCs count in both cytotoxic drugs denoted with asterisks (*p*< 0.001). **i** For haemoglobin, no significant difference was found across all groups except the unconjugated form of FGFR1i denoted with asterisks (*p*< 0.001). (*n*=13)

In the development of novel pharmacological agents, tolerability and therapeutic efficacy are the two most critical factors. After evaluating the therapeutic efficacy of FGFR1i-AuNSs, we examined the treated hamsters for signs of damaging side effects. At first, we monitored different parameters, including weight loss, diarrhea, and alopecia throughout the study period. We did not observe any signs of toxicity after administration of FGFR1i- 5Fu- or CPT-AuNSs. Nine out of the 13 hamsters treated with CPT showed partial alopecia (Fig. 6a). We examined the rest of the HBPC aside from the tumor mass which showed a normal thin corrugated lining epithelium with some focal thinking as a reaction to the DMBA application. Microscopic examination of liver and renal tissue isolated from animals treated with the nanoconjugates showed normal liver and renal tissue architecture. We did not note any signs of toxicity nor AuNSs precipitation in these organs. (Fig. 6b). Areas of glomerular shrinkage were seen in kidney sections after treatment with CPT and 5Fu. These hamsters also showed hydropic degeneration of the hepatocytes with focal necrosis in the central vein zone in liver sections (Fig. 6c-e).

Finally, we assessed the complete blood count through a series of drawn blood samples taken over time to monitor RBC, WBC and platelet counts as a proxy of the animal’s overall health (Fig. 6f, g, i). Multiple comparison tests between complete blood (WBCs, RBCs, platelets, and hemoglobin) counts in our study groups revealed no significance difference between untreated and PEG-AuNSs treated animals which falls within the normal range. This was true across all the time intervals (Fig. 6f-i). Animals treated with drug nanoconjugates did not show any significant change in their WBCs, RBCs, and hemoglobin counts at 1 h, 24 h, 48 h, 1 week, and 4 weeks post-treatment (*p*> 0.999). Regarding platelets count (Fig. 6f), CPT- and 5Fu- AuNSs caused thrombocytopenia one week after treatment. While FGFR1i-AuNSs induced a significant thrombocytosis early in the treatment course at the 1-h time point, which was spontaneously corrected afterwards. Interestingly, free FGFR1i induced significant thrombocytosis persisted until the last time point (4 weeks post-treatment) (*p*< 0.01). For RBCs and WBCs values, there were no significant differences between blood count changes induced by the drug nanoconjugates throughout the whole period (p> 0.999) **(**Fig. 6g, h**)**. However, FGFR1i-, CPT-, and 5Fu-AuNSs induced comparable blood cell readings at all time points. Cytotoxic drugs, free forms of 5Fu and CPT induced a generalized pancytopenia after 1 week of injections. Meanwhile, the unconjugated FGFR1i induced significant anemia starting from the 48-h point interval to 4 weeks post-treatment (Fig. 6i).

## Discussion

It is widely accepted that AuNSs play a crucial role in improving the therapeutic efficiency of anticancer drugs through active targeting and enhanced cellular uptake, thus drug delivery [37]. Studies using single-nanoconjugate system concluded that cellular response and clinical outcome is strictly tailored by the mode of action and pharmacokinetics of that drug conjugated to the AuNSs [38]. Given that studies leading to such conclusions used single nanoconjugates, one could speculate that ruling out the therapeutic potential of AuNSs requires testing different drugs conjugated to similar sized and shaped AuNSs using the same chemical linker. We conducted this study to compare the cellular responses and in vivo clinical outcomes when treating HBPC with conjugated anticancer agents with different mode of actions (CPT-, 5Fu-, FGFR1i- nanoconjugates). Drugs were loaded on the surface of similar sized and shaped AuNPs using the same conjugation techniques as well as the same surface coverage ratio. The nanoconjugates were found to take the same route, starting from being injected to the animal’s vascular system until they induced tumor shrinkage, thereby enhancing survival rate. Upon intraperitoneal injection of AuNSs, the covalently bonded PEG molecules prevented opsonization and subsequent elimination from the blood stream [6]. Once the initial obstacle is bypassed, RGD peptide, also covalently bonded to the AuNSs, actively targets oral cancer cells but not the normal mucosa. RGD targets αvβ6 surface integrins expressed on oral cancer cells [7]. This precise navigation system has proven to be more favorable than PEG-AuNSs alone as previously published by our group [2].

Due to tumor’s acidic pH as well as lysosomal structures, drugs were released via the breakage of pH-sensitive hydrazine bond and subsequently bind to their intracellular targets. Our findings show that AuNSs enhanced tumor penetration and cellular uptake of the drugs compared with their free forms. As expected, AuNSs are the key players in determining cellular uptake rates and not the drugs’ affinity to their targets. This was confirmed by the lack of FGFR1 expression in tumor tissue sections treated with the FGFR1i-AuNSs. Similarly, 5Fu- and CPT- molecules were released by lysosomal acidic pH and consequently localized in the nucleus. This was confirmed by their autofluorescence colocalization with the nucleus, following a previously published concept [39]. Counterintuitively, tumor volumes data suggests that FGFR1i-AuNSs induced higher tumor reduction rates than potent 5Fu- and CPT nanoconjugates. Meanwhile, in normal keratinocytes, FGFR1 is located on the cell surface, however, as reported by Nguyen et al in oral squamous cell carcinoma, FGFR1 is translocated to the cytoplasm, nuclear membrane and nucleus [12]. He observed that in well diff SCC, FGFR1 is expressed in the cytoplasm however the nucleus showed strong FGFR1 expression in poorly diff SCC. His findings confirm that FGFR1i is targeting cytoplasmic and/or nuclear FGFR1 and not the surface receptor in OSCC. Moreover, our confocal results confirmed Nguyen’s findings since FGFR1 fluorescence activity was noted mainly in the cytoplasm. This confirms that RGD was the sole exclude any help from a surface receptor and depending solely on RGD as a targeting moiety like in 5Fu-AuNS and CPT-AuNS treated groups. This suggests that despite being a small molecule inhibitor rather than a potent chemotherapeutic drug, FGFR1i induced the most favorable tumor suppression results once conjugated to AuNPs. Thus, AuNPs play a significant role in magnifying the inhibitory effect of a conjugated molecule rather than being a vehicle.

Given the fact that CPT and 5Fu localize in the nuclei, we assumed that uploading equal number of moieties to the same sized AuNPs will result in equal nuclear uptake. Quantifying 5Fu- and CPT- nuclear uptake in tumor cells show a significant increase in the nuclear localization of CPT compared with 5Fu despite comprising equal surface coverage of AuNPs (55%). However, this is not an indication of an enhanced CPT nuclear uptake, but we speculate that the autofluorescence of 5Fu complex is especially true in the non-catabolic form. Once 5Fu molecule is converted to its active metabolites, they lose the autofluorescence signals giving a false impression of decreased nuclear uptake.

Irrelevant of their mode of action, anti-cancer agents halt cell cycle progression inducing tumor cells to arrest and subsequently trigger cell death pathways. In our work, we were only interested in the downstream effects of the nanoconjugates on the cell cycle machinery and not the cell death kinetics. Flow cytometry and cell cycle data shows that 5Fu- and CPT- induced a similar ratio of S-phase cell cycle arrest as nanoconjugates and in their free forms. CPT and 5Fu inhibit topoisomerase I and thymidylate synthase activities, respectively during DNA replication. There was no additional effect of AuNSs on the cell cycle indicating that their primary role is to deliver drugs to their designated destination at least when bound to drugs such as CPT and 5Fu [2]. Tumor cells treated with free or conjugated FGFR1i molecules exhibited a significant increase in the population of cells arrested in G1-phase compared with saline treated cells. This is due to the FGFR1i mode of action which inhibits MAPK pathway, thus arresting tumor cells in early G1-phase. Interestingly, FGFR1i-AuNSs induced significant sub-G1 cell population indicative of cellular apoptosis. This is likely due to the enhanced potency of the inhibitor in its conjugated form [2, 40]. However, further studies are required to validate and understand the apoptotic effect triggered by FGFR1i-AuNSs on tumor cells. Cell cycle analysis data indicate that potent cytotoxic drugs similar to CPT and 5Fu efficiently induce cell cycle arrest with/without being conjugated to AuNSs. On the other hand, conjugating AuNSs with small molecule inhibitors such as FGFR1i significantly enhance their effect on cell cycle progression compared with their free forms [2].

While the variability in cell cycle arrest patterns induced by 5Fu-, CPT- and FGFR1i- AuNSs was expected, we wondered whether it would have similar functional impact on the proliferative activity/rates of tumor cells. To answer this question, we measured the proliferative activity of tumor cells by staining tumor tissue sections isolated from different animal groups with anti- Ki67 and anti-PCNA markers. To our surprise, there was no significant difference in the proliferation rate of tumor cells treated with CPT-, 5Fu- and FGFR1i- AuNSs. Cell cycle arrest in S- or G1- phase induced similar cessation of proliferation rates [41]. However, drug nanoconjugates significantly halted tumor cells proliferation compared with the free forms of the drugs. This suggests that the enhanced cellular uptake and drug delivery by the AuNSs are more crucial than the drug’s mode of action in ceasing the proliferation of cancer cells [8]. Despite the fact that tumor cells primarily form tumor tissue, it would be naïve to expect that tumor cell growth necessarily reflects tumor growth rate [42]. Tumor growth rate is suggested to be an outcome of complex interactions between tumor cells and their microenvironment, including fibroblasts, and blood vessels. Despite conferring similar proliferative rates, FGFR1i-AuNSs induced the highest tumor reduction rates (− 64%) followed by 5Fu- AuNSs (~− 45%). CPT-AuNSs induced significantly lower tumor reduction rates compared with the 5Fu- and FGFR1i- AuNSs despite showing similar proliferation activity on the tumor cell level, as confirmed by the IHC data. This can be explained by the non-autonomous cellular effects induced by FGFR1i [43].

Off-target side effects of anti-cancer agents such as CPT, 5Fu, and FGFR1i are well discussed in the literature focusing mainly on hemopoietic stem cell senescence, elevated liver and renal function tests, and alopecia [14, 44–46]. Our findings confirm that conjugating anticancer drugs to AuNSs significantly minimized the deleterious impact of otherwise free drugs molecules on the bone marrow. FGFR1i-AuNSs showed a slight thrombocytosis only at 1 h which was spontaneously corrected afterwards. While 5Fu- and CPT- AuNSs showed minimal thrombocytopenia 48 h after treatment, we considered that minor changes in the blood parameter counts are due to gradual increase in the blood pH throughout the carcinogenesis process leading to dissociated to PH-sensitive linker between the NSs and different drugs prior reaching the target area [47].

## Conclusions

In conclusion, despite having different modes of action, CPT-, 5Fu-, FGFR1i- nanoconjugates seemed to differently regulate cell cycle response at the cellular level. Such variability in the cell cycle response did not have any significant impact on the overall clinical impact. Our data indicates that the cellular biological events do not predict the outcome seen in our in vivo model. Furthermore, our results suggest that AuNSs selectively enhances the therapeutic effect of small molecule inhibitors such as FGFR1i than potent anticancer drugs. These findings suggest that AuNSs play a bigger role than being drug vehicles. Future studies are required to better understand the underlying mechanism.

## Data Availability

All data included in this current study are available from the corresponding author upon request.

## Abbreviations

AuNPs: Gold nanoparticles
AuNSs: Gold nanospheres
5Fu: 5-Fluorouracil
CPT: Camptothecin
FGFR1i: Fibroblastic growth factor receptor 1
PCNA: Proliferating cell nuclear antigen
IHC: Immunohistochemistry
PEG: Polyethylene glycol
RGD: Arginyl-glycyl-aspartic acid (Arg-Gly-Asp)
MAPK: Mitogen-activated protein kinase
HBPC: Hamster buccal pouch carcinoma
OSCC: Oral squamous cell carcinoma
DMBA: 7, 12 dimethylbenz [a] anthracene carcinogen
Hr: Hour
